# Precise Construction of Dual-Promising Anticancer Drugs Associated with Gold Nanomaterials on Glioma Cancer Cells

**DOI:** 10.1155/2023/8892099

**Published:** 2023-10-25

**Authors:** P. Baby Shakila, Abdurahman Hajinur Hirad, Abdullah A. Alarfaj, Samer Hasan Hussein-Al-Ali, Beza Mulugeta

**Affiliations:** ^1^Department of Biochemistry, Vivekananda College of Arts and Sciences for Women, Tiruchengode 637205, Tamil Nadu, India; ^2^Department of Botany and Microbiology, College of Science, King Saud University, P.O. Box 2455, Riyadh 11451, Saudi Arabia; ^3^Department of Chemistry, Faculty of Science, Isra University, Amman 11622, Jordan; ^4^Department of Food Science and Postharvest Technology, Haramaya Institute of Technology, Haramaya University, Dire Dawa, P.O. Box 128, Ethiopia

## Abstract

Multiple chemodrugs with nanotechnology have proven to be an effective cancer treatment technique. When taken combined, cabazitaxel (CTX) and cisplatin (PT) have more excellent cytotoxic effects than drugs used alone in the chemotherapy of several different cancers. However, several severe side effects are associated with using these chemotherapy drugs in cancer patients. Gold nanomaterials (AuNMs) are promising as drug carriers because of their small diameter, easy surface modifications, good biocompatibility, and strong cell penetration. This work aimed to determine the CTX and PT encapsulated with AuNMs against human glioma U87 cancer cells. The fabrication of the AuNMs achieved a negative surface charge, polydispersity index, and the mean sizes. The combined cytotoxic effect of CTX and PT bound to AuNMs was greater than that of either drug alone when tested on U87 cells. The half inhibitory concentration (IC_50_) values for free PT were 54.7 *μ*g/mL (at 24 h) and 4.8 g *μ*g/mL (at 72 h). Results acquired from the MTT assay show cell growth decreases time- and concentration-dependent AuNMs, free CTX, free PT, and AuNMs@CTX/PT-induced cytotoxicity and, ultimately, the cell death of U87 cells via apoptosis. The biochemical apoptosis staining techniques investigated the cells' morphological changes of the cells (acridine orange and ethidium bromide (AO-EB) and nuclear staining (DAPI) techniques). The AO-EB and nuclear staining results reveal that the NPs effectively killed cancer cells. Furthermore, the flow cytometry analysis examined the mode of cell death. Therefore, AuNMs@CTX/PT has excellent potential in the cancer therapy of different cancer cells.

## 1. Introduction

Globally, cancer is a significant public health issue. Every year, more than eleven million people are diagnosed with different cancers [1–5]. With an annual prevalence of 6.6 per 100,000 people worldwide, gliomas are the most widespread primary tumours of the central nervous system (CNS). They arise from glial cells (ependymal, oligodendrocytes, or astrocytes) [6–8]. Gliomas are the most common malignant brain tumour, around 80% in total [9]. WHO categorizes gliomas into four distinct stages. With its high recurrence rate and widespread tumour cell infiltration into the brain parenchyma, glioblastoma multiforme (GBM) is the most common and dangerous form of malignant gliomas [10–12]. There are 3.19 new cases of GBM for every 100,000 people in the United States. Patients treated according to current guidelines had a median survival duration of 14.6 months after diagnosis and a 5-year survival rate of 9.8% [13]. The current treatment protocol includes tumour resection surgery, radiation therapy (RT), and temozolomide chemotherapy as adjuvant therapy [14]. However, the infiltrative high-grade gliomas, which invade vital brain structures, and the lack of clarity in tumour margins pose challenges to the complete resection of gliomas [15]. The invasive glioma cells frequently form secondary tumours within a few centimetres of the tumour site after surgery. However, most anticancer drugs cannot cross the blood-brain barrier (BBB); hence, chemotherapy only shows limited clinical advantages [16]. Tight connections between endothelial cells, pericytes, and the end foot of astrocytes make up the BBB and control the flow of chemicals into the brain from the circulatory system [17]. So, most therapeutic and diagnostic agents do not get too high enough concentrations in the tumour microenvironment. Two examples of intervention tactics are temporary rupture of the direct drug delivery and BBB by intracerebral injection [18]. However, they are both extraordinarily invasive and unsuitable for long-term treatment. Furthermore, reducing chemotherapy's effectiveness are the drugs' short half-lives and poor solubility in blood circulation [19]. However, the cumulative dosage of radiation that may be securely delivered to keep the accompanying toxicities in the normal tissues acceptable limits the extent to which RT can be applied [20].

Considering the limitations of current glioma diagnosis and treatment methods, nanomedicine has emerged as a potentially effective replacement [21–23]. Gold nanomaterials (AuNMs) have garnered considerable interest among the numerous systems utilized due to their tunable surface functions, unique optical properties, synthetic adaptability, and biocompatibility [23]. The high surface-to-volume ratio of nanomaterials allows for their rapid uptake of imaging contrast agents, drugs, and biomolecules. Therefore, AuNMs have been used for many theranostics applications, such as imaging (e.g., surface-enhanced Raman scattering, magnetic resonance, photoacoustic, and computed tomography), drug administration (e.g., siRNAs and proteins), and drug therapy (e.g. RT, and photothermal therapy) [24–26]. Generally, theranostics is a bridge between diagnostic and therapeutic approaches. In imaging-guided therapy, AuNMs enable microsurgical excision of tumours at a submillimeter spatial resolution, monitor drug administration, and delineate glioma margins with great sensitivity [27–29]. Several research groups reported drug-loaded gold nanomaterials for treating different cancer cells, which shows severe adverse effects [23–25]. Recently, Wan et al. have described the anticancer effects of docetaxel conjugated AuNMs, where the samples show greater cytotoxicity against the HepG2 liver cancer cell line and exposed to have enhanced bioavailability at the tumor site [30].

Castration-resistant prostate cancer can be treated with cabazitaxel (CTX), a second-generation semisynthetic taxanelicensed by the FDA and the European Drugs Agency [31]. The first-generation taxane treatments (such as paclitaxel and docetaxel) have limited utility [32]. Docetaxel and paclitaxel are high-affinity substrates for ATP-dependent multidrug-resistant pumps. Because of difficulties with endothelial influx/efflux ratios, tumour-induced activation of multidrug-resistant pumps decreases effective concentrations of therapeutic and drug penetrations around the BBB [33]. The reduced attraction of multidrug-resistant pumps for cabazitaxel compared to paclitaxel and docetaxel suggests that it will be more effective against brain malignancies [34]. Recent studies have also demonstrated in vivo that cabazitaxel is widely distributed throughout the brain and may be taken up by BBB endothelial cells [35, 36].

Glioma is treated with cisplatin, also known as *cis*-diamminedichloroplatinum (II), one of the most effective chemotherapeutics for the treatment of testicular, ovarian, neck, and head tumours and in neoadjuvant or adjuvant therapy of other tumours [37]. Cisplatin primarily interacts with DNA purine bases to inhibit tumour cell growth and induce death, producing DNA-protein and DNA-DNA interstream and intrastrain crosslinks [38–40]. Combining cisplatin with other chemotherapeutics, such as Gleevec, Paclitaxel, or Gemcitabine, has been shown to sensitize many tumour cell types to cisplatin, hence overcoming cisplatin resistance and toxicity [41]. Metformin has been shown to enhance the response to cisplatin-free treatment and potentate cisplatin-mediated killing of epithelial cancer cells in vitro, making it promising for combining cisplatin-based therapy [42]. However, research into the cytotoxic efficacy of the metformin/cisplatincombination in treating various cancer cell lines is still in its infancy [43].

**Figure 1:**
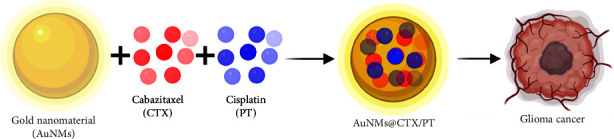
Graphical representation of cabazitaxel (CTX) and cisplatin (PT) associated with gold nanomaterials (AuNMs) fared against human glioma U87 cancer cells.

Researchers are currently focusing on bettering glioma cancer treatments by exploring a variety of therapy methods, with an emphasis on nanotechnology. Gold nanomaterials (AuNMs) have many applications in material science and catalysis [44–46]. They also have significant benefits, such as the ability to be easily scaled down to sizes similar to biomolecules, making them more accessible to biological systems [47]. The anti-inflammatory and antitumour properties of AuNMs have piqued scientific interest in their potential as drug carrier systems [48]. In addition, AuNMs can transport chemotherapeutic drugs to their intended sites of action, maximizing therapy efficacy while minimizing side effects. It is worth noting that studies combining CTX and PT were not discovered, although several studies were reported of using either drug alone in conjunction with gold nanomaterials (Figure 1). This work examined the cytotoxicity of AuNMs coupled with CTX and PT on glioma cells by synthesis, characterization, and in vitro cytotoxicity assessment. In addition, the biochemical apoptosis staining techniques investigated the cells' morphological changes of the cells (AO-EB and DAPI techniques). Furthermore, the flow cytometry analysis examined the mode of cell death. Therefore, AuNMs@CTX/PT has excellent potential in the cancer therapy of different cancer cells.

## 2. Experimental Section

### 2.1. Methods and Materials

Cisplatin (PT), cabazitaxel (CTX), chloroauric acid (HAuCl_4_), and sodium citrate (Na_3_C_6_H_5_O_7_) were bought from Aladdin Co., Ltd. 3-(4,5-dimethylthiazol-2-yl)-2,5-diphenyltetrazolium bromide (MTT), dimethyl sulfoxide (DMSO), and phosphate-buffered saline (PBS) were obtained from Sigma-Aldrich (USA). Acridine orange/ethidium bromide (AO/EB) and 4′,6-diamidino-2-phenylindole (DAPI) were ordered from Thermo Fisher Scientific, Inc. (USA). All the chemicals and reagents were used without further purification.

### 2.2. Fabrication of AuNMs

The previously reported procedure was successfully used to construct AuNMs [49]. Na_3_C_6_H_5_O_7_ was used as a reductant and a stabilizer in the chemical reduction of the metal precursor HAuCl_4_. Under constant stirring, a 1 mM HAuCl_4_ solution was heated to 95°C. By gradually adding 38 mM Na_3_C_6_H_5_O_7_ solution (at 50°C), 5 mL of HAuCl_4_ was converted into AuNMs. Once a solution of the desired purple colour was formed, the reaction was allowed to cool to ambient temperature.

### 2.3. Construction of AuNMs Containing Drugs

With constant mixing, drug association was carried out with a fixed concentration of CTX or PT in AuNMs solution. A DMSO solution containing 10 *μ*g/mL of CTX and 100 *μ*g/mL of PT was prepared. To facilitate better drug-AuNMs interaction, the solution was agitated at 100 rpm for 24 h (at room temperature). The free drug was extracted from the AuNMs by centrifuging them at 10,000 rpm for 30 minutes.

### 2.4. Characterization of the Nanomaterials

Samples were prepared by depositing dilute nanoparticle solutions onto carbon film-coated copper grids. TEM images were acquired with an FEI Tecnai F20 TEM (Hillsboro, OR) operating at 200 kV. Nanomaterials were synthesized and then lyophilized to obtain dry samples. The samples were mixed into a KBr pellet at 0.2 wt%. The FTIR spectra of the various nanoparticle samples were obtained using Nicolet 6700 FTIR Spectrometer (Thermofisher, Waltham, MA). The nanomaterial's hydrodynamic size and zeta potential were measured through dynamic light scattering (DLS) via Zetasizer Nano ZS90 (Malvern Instruments, UK). The absorbance was measured at 570 and 630 nm using a microplate reader (Thermo Fisher Scientific, Waltham, MA). UV-vis absorption spectra were recorded using a UV-visible spectrophotometer (Evolution 220, Thermo Fisher Scientific). Cell images were taken using a fluorescence microscope (Olympus CKX53, Japan).

### 2.5. Quantification of CTX and PT on the AuNMs

UV-vis spectroscopy was used to identify the CTX and PT concentration range in DMSO standard solutions from 0.1 to 5 *μ*g/mL and 10 to 100 *μ*g/mL for the drug quantification tests on the AuNMs. Absorbance was checked at 213 nm (Brix) and 229 nm (CTX) (PT). A calibration curve was obtained for all drugs with an R2 greater than 0.98. The amount of AuNMs precipitated after being centrifuged at 13,000 rpm for 30 minutes was measured. Previous approaches were used to quantify drugs by percentage.

### 2.6. Anticancer Activity (MTT Assay)

The human U87 glioma cancer cells were acquired from the Cell Bank of the Chinese Academy of Sciences (Shanghai, China). U87 cells were cultured in the RPMI 1640 medium supplemented with 10% fetal bovine serum, 100 U of penicillin, and 100 *μ*g/mL streptomycin in a hypoxic chamber (5% CO_2_) at 37°C.

The MTT assay assessed the anticancer efficacy of AuNMs, free CTX, free PT, AuNMs@CTX/PT, and in U87 glioma cancer cell lines. After 24 and 72 h of incubation in a 96-well, the cells (2 × 10^3^/each well) were treated with varying doses of the samples. After 24 and 72 h, MTT solution (5 mg/mL) was poured into each well at 10 *μ*L. An additional 4 hours were spent incubating the cells. The formazan crystals were dissolved by removing the excess medium from each well and replacing it with 100 *μ*L of acidified isopropanol. Microplate spectrophotometer (BioTek, USA) readings at 570 nm were used to compile optical density values. Inhibitory concentration (IC_50_) values were determined using GraphPad Prism 8.0 [50–52].

### 2.7. Apoptosis Staining

U87 cells were planted at a density of 5 × 10^3^ cells/well in 24-well plates and cultured overnight. AuNMs, free CTX, free PT, and AuNMs@CTX/PT to induce apoptosis in U87 cells were evaluated by the AO/EB double staining method. U87 cells were cultured with AuNMs, free CTX, free PT, and AuNMs@CTX/PT at IC_50_ concentrations. The plates were incubated for 24 and 72 h at 37°C. After 24 h of incubation with an AO/EB staining solution for 5 min, the apoptosis of the U87 cells was analyzed using a fluorescence microscope [53].

Nuclear morphological changes, such as condensation or fragmentation, associated with apoptosis can be assessed by DAPI labelling. U87 cells were seeded at a density of 5 × 10^3^ cells/each well in 24-well plates and incubated overnight. In this assay, U87 cells were incubated of AuNMs, free CTX, free PT, and AuNMs@CTX/PT and incubated for 24 and 72 h at 37°C. Following incubation, the cells were washed twice with PBS, fixed with 4% paraformaldehyde, and rehydrated in 70% ethanol. After incubation with DAPI staining solution and after incubation for 5 min, the apoptosis of the U87 cells was analyzed using a fluorescence microscope [54–56].

U87 cells were planted at a density of 5 × 10^3^ cells/well in 96-well plates and cultured overnight. After that, the medium was changed with a fresh medium (pH 7.2) including AuNMs, free CTX, free PT, and AuNMs@CTX/PT for 24 and 72 h at IC_50_ concentration. After further incubation for 24 and 72 h, the cells were rinsed thrice with PBS, collected, and dyed with the Annexin V-FITC/PI assay kit. The quantitative apoptosis assay was investigated by flow cytometric investigation [57].

### 2.8. Statistical Analysis

The data shown here were presented as the mean ± standard deviation (SD). The significance of the deviation was verified by one-way ANOVA, which was significant when *P* < 0.05 and very substantial when *P* < 0.01.

## 3. Results and Discussion

### 3.1. Physicochemical Characterization

**Figure 2:**
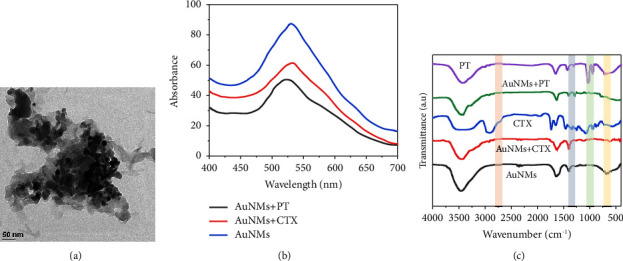
Morphological characterisation of cabazitaxel (CTX) and cisplatin (PT) associated with gold nanomaterials (AuNMs). (a) TEM image of AuNMs@CTX/PT. (b) UV-vis analysis of free AuNMs and AuNMs associated with CTX and PT. (c) FTIR analysis of free AuNMs, PT, CTX, and AuNMs associated with CTX and PT.

UV-vis analysis assessed the AuNMs' optical characteristics by monitoring for wavelength shifts caused by drug association. The maximum absorbance wavelength of free AuNMs, which is 526 nm for AuNMs of size 20 nm, was not changed by CTX, as shown in Figure 2(b). Slight wavelength shifts were shown when AuNMs were mixed with PT, indicating a more vital chemical interaction between the carboxylic acid on the AuNMs' surface and the PT. This finding is consistent with drug quantification results reported by UV-vis spectroscopy, which also showed a high level of interaction between AuNMs and PT (88% ± 5%). A combined percentage calculation of AuNMs and CTX yielded 48 ± 5%.

The free AuNMs employed in this work had an average diameter of 20 nm, confirmed by transmission electron microscopy (TEM) (Figure 2(a)). This confirms the accuracy of the UV-vis analysis. As anticipated, the average diameter of AuNMs showed a considerable increase after association with various drugs, indicating that the drugs formed a layer on the surface of the AuNMs. While the polydispersity index for PT-related AuNMs was >0.2, it was narrower for free AuNMs and AuNMs linked with CTX. In addition, drug-AuNMs' interactions changed the charged surface. High zeta potential was observed in the AuNMs, correlated with enhanced colloidal stability. As a result, we can infer that the AuNMs have a stable drug coating and perform well in stability tests. Transmission electron microscopy (TEM) and ultraviolet-visible spectroscopy all corroborated the findings that the drugs had a connection to AuNMs. The mica surface, which the nanomaterials were easily spread throughout, was hydrophilic.

We used Fourier transform infrared spectroscopy (FTIR) to analyze the drug's chemical structure and investigate its potential interactions with AuNMs.  Figure 2(c) displays the peak properties of AuNMs, which coincide with the COO's symmetric and asymmetric stretching.

The FTIR spectra of free PT, AuNMs@PT, free CTX, AuNMs@CTX, and AuNMs were noted in the spectral regions at 4000–400 cm^−1^ as demonstrated in Figure 2(c). The FTIR spectra of AuNMs demonstrated the -OH symmetric stretching vibration at 3459.14 cm^−1^and 3268.54 cm^−1^, representing the presence of the alcoholic group. The presence of the C-H bond was noted at 2845.96 cm^−1^, and the presence of the C=O bond was recorded at 1610.19 cm^−1^, 1593.41  cm^−1^. The bands at 1427.45 cm^−1^ and 1374.57 cm^−1^ fitted to C-C-C stretching, while the wave number 1267.42 cm^−1^ to 1072.47 cm^−1^ indicated the carboxylic C-O groups. The combination of AuNMs and PT showed the signature peaks of AuNMs and the stretching bands at about 3300 and 1297 cm^−1^. However, no additional peaks were observed. Furthermore, no new peaks were observed in the AuNMs@CTX combination, indicating no chemical interaction between the drugs and the AuNMs.

### 3.2. In Vitro Cytotoxicity Investigation

**Figure 3:**
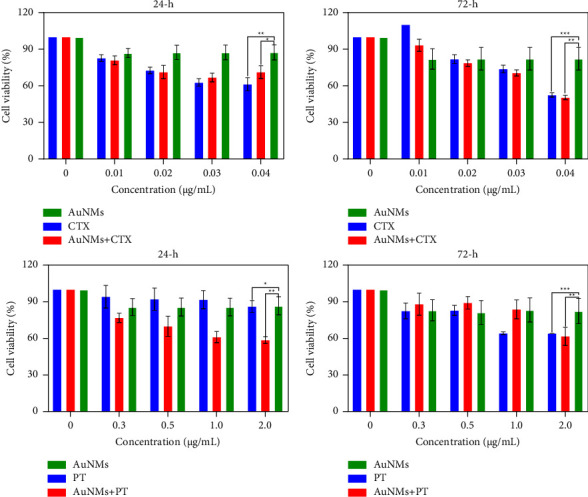
In vitro cytotoxicity of the AuNMs, free CTX, free PT, AuNMs@CTX, and AuNMs@PT was investigated by the MTT assay using U87 glioma cancer cells for 24  and 72 h. Data were expressed as means ± SEM (*n* = 3). (^*∗*^*P* < 0.05, ^*∗∗*^*P* < 0.01, ^*∗∗∗*^*P* < 0.001).

**Figure 4:**
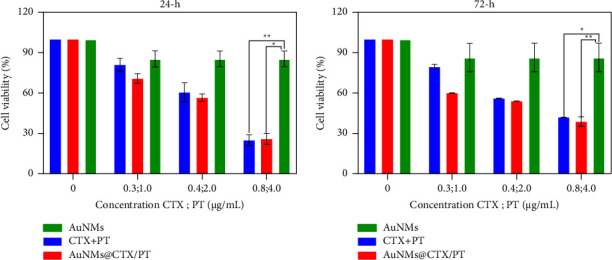
In vitro cytotoxicity of the AuNMs, CTX + PT, and AuNMs@CTX/PT was investigated by the MTT assay using U87 glioma cancer cells for 24  and 72 h. Data were expressed as means ± SEM (*n* = 3). (^*∗*^*P* < 0.05, ^*∗∗*^*P* < 0.01).

As shown in Figure 3, we tested the cytotoxicity of AuNMs, free CTX, free PT, and AuNMs@CTX/PT in U87 cells at varying doses and incubation durations (24 and 72 h). After 24 h (Figure 3) and 72 h (Figure 3) of exposure, U87 cells treated with 0.01, 0.02, 0.03, and 0.04 *μ*g/mL of free CTX and AuNMs@CTX revealed a substantial decrease in U87 cell viability (Figure 3). Both free CTX and AuNMs@CTX reduced cell viability in a concentration-dependent way after 72 h of exposure. Still, there was no other significant difference between the groups. It was shown that although free CTX caused a 27% reduction in cells at a dose of 0.03 *μ*g/mL, AuNMs@CTX caused a 32% reduction. Cell viability decreased by 47% when exposed to free CTX and 50% when exposed to AuNMs@CTX at a dose of 0.04 *μ*g/mL. Despite not having a more potent cytotoxic impact, AuNMs@CTX in the 24 h exposure period demonstrated significant effects compared to free CTX at both the evaluated 0.01 and 0.02 *μ*g/mL dosages. At a 0.01 *μ*g/mL concentration, free CTX reduced cell viability by 18%, whereas AuNMs@CTX reduced viability by 20%. Free CTX decreased cell viability by 29% and AuNMs@CTX by 32% at a 0.02 *μ*g/mL concentration. IC_50_ values for the suppression of U87 cell viability by free CTX and its combination with AuNMs were similar across the tested groups. At the concentration employed in all the studies (10 *μ*g/mL), free AuNMs exhibited no appreciable cytotoxic effect on the U87 cells. U87 cells exposed to AuNMs@PT for 24 h demonstrated a more potent cytotoxic effect than those exposed to free PT, resulting in a concentration-dependent decrease in cell viability (Figure 4). In comparison to the concentrations of 0.3, 0.5, 1.0, and 2.0 *μ*g/mL, the reduction in cell viability for free PT was 9, 5, 8, and 15%, while the reduction in cell viability for AuNMs@PT was 27, 34, 37, and 44%. The half inhibitory concentration (IC_50_) values for free PT were 54.7 *μ*g/mL (at 24 h) and 4.8 g *μ*g/mL (at 72 h). The IC_50_ value for PT in combination with AuNMs after 24 h of incubation was substantially lower than that of free PT. The MTT assay results suggested that CTX and PT treatment combined with AuNMs had a more significant cytotoxic effect.

### 3.3. Apoptosis Investigation

**Figure 5:**
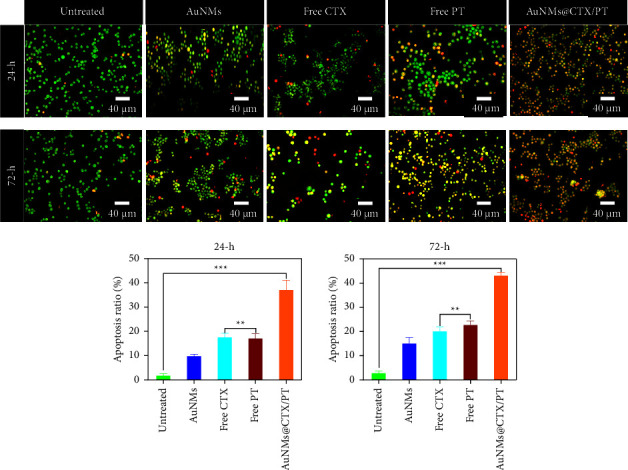
The morphological changes of the AuNMs, free CTX, free PT, and AuNMs@CTX/PT. AO-EB staining of AuNMs, free CTX, free PT, and AuNMs@CTX/PT treated with U87 glioma cancer cells for 24 and 72 h. Scale bar 40 *μ*m. Data were expressed as means ± SEM (*n* = 3). (^*∗∗*^*P* < 0.01, ^*∗∗∗*^*P* < 0.001).

**Figure 6:**
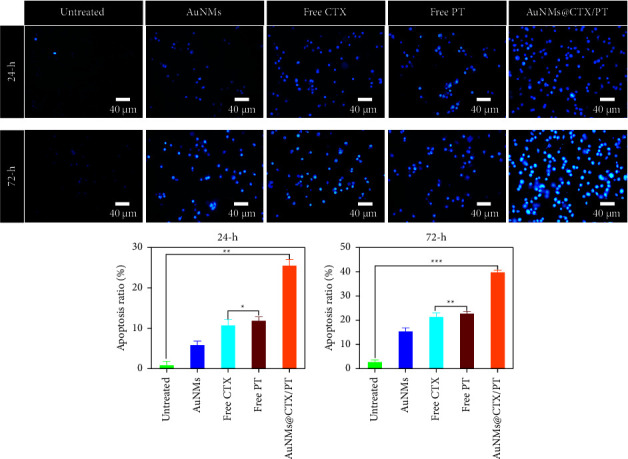
The nuclear morphology changes of the AuNMs, free CTX, free PT, and AuNMs@CTX/PT. DAPI staining of AuNMs, free CTX, free PT, and AuNMs@CTX/PT treated with U87 glioma cancer cells for 24 and 72 h. Scale bar 40 *μ*m. Data were expressed as means ± SEM (*n* = 3). (^*∗*^*P* < 0.05, ^*∗∗*^*P* < 0.01, ^*∗∗∗*^*P* < 0.001).

The physical feature of chromatin condensation in the stained nucleus and the differential uptake of fluorescent DNA binding dyes acridine orange and ethidium bromide are utilized in the acridine orange (AO)/ethidium bromide (EB) double staining approach [58–62]. AuNMs, free CTX, free PT, and AuNMs@CTX/PT toxicity led to a decrease in viable cells (VN) and an increase in early apoptotic (VA), late apoptotic (NVA), and necrotic cells (NVN). AO/EB labelling tracked the apoptosis rate in U87 glioma cancer cells after they were treated with AuNMs, free CTX, free PT, and AuNMs@CTX/PT (Figure 5). The data show that both early and late apoptotic cells are present in IC_50_-treated cells with 24 and 72 h (Figure 5). Green with numerous orange spots indicated chromatin condensation and nuclear disintegration in early apoptotic cells. Orange staining of late apoptotic cells was indicative of condensed and shattered nuclei. The untreated (control) cells kept their green colour and regular shape. Nuclear staining with DAPI was conducted to provide context for the findings (Figure 6). Figure 6 demonstrates that after staining with DAPI, cells treated with 24 and 72 h to the IC_50_ concentrations of AuNMs, free CTX, free PT, and AuNMs@CTX/PT had a higher number of nuclear fragmentations than the control group. The findings corroborated those from the dual staining method.

**Figure 7:**
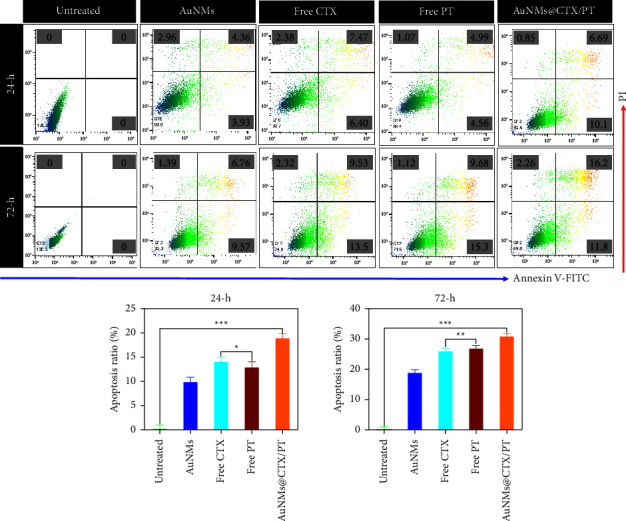
The apoptosis of the AuNMs, free CTX, free PT, and AuNMs@CTX/PT were confirmed by flow cytometry analysis. The U87 glioma cancer cells were stained with Annexin V-FITC and PI for 24 and 72 h. Data were expressed as means ± SEM (*n* = 3). (^*∗*^*P* < 0.05, ^*∗∗*^*P* < 0.01, ^*∗∗∗*^*P* < 0.001).

The three main types of cell death are apoptosis, autophagy, and necrosis. An apoptosis kit including PI and FITC-labeled annexin-V dye was used to examine the cancer cells' apoptosis [14]. During the apoptosis stage, phosphatidylserine (PS) exposed outside the cell can be highly explicitly bound by the annexin V dye. According to the findings, the U87 cells' apoptosis rate increased following stimulation with AuNMs, free CTX, free PT, AuNMs@CTX/PT, and AuNMs@CTX/PT. Apoptosis also increased with the concentration of the AuNMs@CTX/PT (Figure 7). The cells were exposed for 24 and 72 h to the IC_50_ concentrations of AuNMs, free CTX, free PT, and AuNMs@CTX/PT to calculate the percentage of apoptotic and necrotic cells. In this case, we discovered that AuNMs@CTX/PT could cause apoptosis in U87 cells. The findings of the flow cytometry analysis demonstrated that the IC_50_ concentration of AuNMs@CTX/PT increased the apoptosis rate in the treated cells (Figure 7).

## 4. Conclusion

These studies show that CTX and PT, in conjunction with AuNMs, can be an effective therapeutic strategy. Nanomaterials' size on the nanometer scale, high colloidal stability, and negative zeta potential were all characteristics. The particle size of gold nanomaterials was about ∼20 nm. Our data demonstrated that the cytotoxic effect of the drugs in combination with AuNMs on U87 cells was greater than that of the CTX and PT drugs. AuNMs@CTX/PT results of cytotoxicity tests in vitro had good biocompatibility. The biochemical apoptosis staining techniques investigated the cells' morphological changes of the cells (AO-EB and nuclear (DAPI) staining techniques). Furthermore, the flow cytometry analysis examined the mode of cell death. Thus, this exploration indicates that the fabricated AuNMs@CTX/PT may deliver an excellent idea for fabrication and improve biocompatible and safe nanomaterials that can carry a wide range of therapeutic agents to treat glioma cancer.

## Data Availability

All data generated or analyzed during this study are included within the article. The raw data shall be made available upon request from the corresponding author.
